# Adrenal Biomarkers of Stress in Transgender and Gender‐Diverse Adolescents

**DOI:** 10.1002/dev.70156

**Published:** 2026-04-19

**Authors:** Simone Coslovich, Stefania Tonetto, Giulia Bragato, Gianluca Tamaro, Alice Fachin, Paolo Dalena, Lorenzo Zandonà, Antonella Fabretto, Egidio Barbi, Gianluca Tornese

**Affiliations:** ^1^ University of Trieste Trieste Italy; ^2^ Institute for Maternal and Child Health—IRCCS “Burlo Garofolo” Trieste Italy; ^3^ SC Laboratorio Unico, Ospedale Maggiore Azienda Sanitaria Universitaria Giuliano Isontina Trieste Italy

**Keywords:** adolescent, adrenal, gender dysphoria, gender incongruence, stress, transgender and gender diverse

## Abstract

Transgender and gender diverse (TGD) adolescents are frequently exposed to minority stress, which may influence the hypothalamic–pituitary–adrenal (HPA) axis during critical developmental windows. Altered cortisol dynamics have been described in populations facing chronic stress, yet evidence in TGD youth is limited. Understanding adrenal function in this context is essential for clarifying potential biological pathways linking social stressors to developmental and health outcomes. In the present study, identifying as TGD serves as an indirect proxy of exposure to minority stressors, which were not directly measured. We conducted a retrospective, case–control study at a tertiary pediatric center, including 48 TGD adolescents and 298 controls referred for evaluation of premature pubarche with nonclassical congenital adrenal hyperplasia excluded. All participants underwent a standard dose synacthen test (SDST; 250 µg tetracosactide iv, sampling at baseline and 60 min, 8:00 a.m.), which assesses adrenal responsiveness to pharmacological ACTH stimulation. Serum cortisol, DHEAS, ACTH, and 17‐hydroxyprogesterone were assayed. Statistical analyses included nonparametric group comparisons, correlations, and multivariable regression adjusting for age and sex assigned at birth. TGD individuals demonstrated significantly higher baseline cortisol levels (293 vs. 214 nmol/L; *p* < 0.001) and a reduced cortisol response to SDST (Δcortisol: 354 vs. 446 nmol/L; *p* < 0.001). In the full sample, basal DHEAS levels were higher in TGD youth (231 vs. 142 nmol/L; *p* = 0.362), whereas the DHEAS‐to‐cortisol ratio did not differ significantly between groups. In an age‐matched subsample (1:2 matching), the DHEAS‐to‐cortisol ratio was significantly lower in TGD adolescents (0.72 vs. 1.03; *p* = 0.004). Multivariate analysis confirmed an independent association between TGD status and higher basal cortisol, lower Δcortisol, and a reduced DHEAS‐to‐cortisol ratio after adjustment for covariates (all *p* < 0.001). Our findings provide preliminary evidence of altered adrenal responsiveness in TGD adolescents, potentially reflecting the biological embedding of minority stress during development. Although exploratory, these results highlight the need for prospective, longitudinal studies integrating psychosocial and neuroendocrine measures to clarify mechanisms linking stress, HPA axis function, and developmental outcomes in gender‐diverse youth.

## 1 Introduction

Transgender and gender diverse (TGD) youth face unique health challenges that extend beyond access to gender‐affirming care (Coleman et al. 2022). The minority stress framework, originating with Brooks (1981) and formalized by Meyer (2003), describes how distal stressors (e.g., enacted stigma, victimization) and proximal stressors (e.g., expectations of rejection, internalized stigma) generate chronic psychological and physiological burdens. Extensions of this model for TGD individuals highlight the unique stressors associated with gender identity and expression, including pressures to conform to cisnormative expectations and threats to safety (Hendricks and Testa 2012; Puckett et al. 2023; Testa et al. 2015), often referred to as “gender minority stress”. These processes not only contribute to mental health disparities but also may influence biological systems involved in stress regulation.

From an embodiment perspective, social adversity becomes biologically embedded through cumulative activation of stress‐related pathways (Chae et al. 2010). A core system involved in this embedding is the hypothalamic–pituitary–adrenal (HPA) axis, which coordinates hormonal responses to everyday and acute stressors. Chronic activation of the HPA axis—common in individuals facing persistent stigma—can lead to altered diurnal cortisol rhythms, attenuated responsiveness to acute challenge, and increased allostatic load (McEwen 1998; Krieger 2010; Juster et al. 2010), which refers to the cumulative physiological burden of adaptation to stress. These studies, including early work by DuBois et al. and more recent summaries of cortisol findings across TGD samples, converge in documenting altered diurnal cortisol patterns in association with gender‐related stressors (DuBois 2012; DuBois et al. 2017; DuBois and Juster 2022; DuBois et al. 2024), together providing a cumulative evidence base on stress‐related HPA axis alterations in TGD adults. Similar alterations have been observed in sexual and gender minority young adults, where minority stressors were associated with heightened morning or evening cortisol levels (Zoccola et al. 2017; Figueroa et al. 2021). Additional work has reported heightened cortisol awakening responses and elevated perceived stress before gender‐affirming treatment (Colizzi et al. 2013). Together, these findings suggest that chronic exposure to stigma may contribute to characteristic patterns of HPA axis dysregulation.

Despite these advances, relatively little is known about HPA axis functioning in TGD adolescents. Adolescence is a sensitive period marked by rapid neuroendocrine changes, including maturation of the HPA axis and activation of the hypothalamic–pituitary–gonadal (HPG) axis (Marceau et al. 2015; Powers and Casey 2015). Pubertal timing and sex‐assigned‐at‐birth differences also shape developmental trajectories of cortisol and adrenal androgen secretion, with youth assigned female at birth typically entering puberty earlier than those assigned male at birth (Biro et al. 2014). These developmental processes heighten sensitivity to social evaluation and may amplify the biological impact of minority stress. Understanding how social stressors intersect with pubertal maturation is, therefore, crucial for elucidating psychobiological pathways linking adversity to health outcomes in TGD youth.

Adrenal androgens—particularly dehydroepiandrosterone sulfate (DHEAS)—play a central role in adolescent stress physiology. DHEAS levels rise markedly during adrenarche and adolescence and exert neuroprotective, immunomodulatory, and anti‐glucocorticoid effects (Kroboth et al. 1999; Gallagher and Young 2002). Because DHEAS and cortisol exert opposing influences, the DHEAS‐to‐cortisol ratio has been proposed as an index of stress‐related resilience versus vulnerability. Lower ratios—reflecting elevated cortisol, reduced DHEAS, or both—are common under chronic psychosocial stress and have been associated with maladaptive emotional and physiological outcomes, including depressive symptoms and immune dysregulation (Buford and Willoughby 2008; Gürpinar et al. 2019; Lennartsson et al. 2022; Kimonis et al. 2019). For these reasons, simultaneous assessment of cortisol, DHEAS, and their ratio provides a more complete characterization of adrenal stress biology, particularly during adolescence.

Although a few studies have examined basal or reactive cortisol responses in TGD adolescents, findings have been mixed, with reports of both heightened and blunted reactivity (Pommier et al. 2019; Kokka et al. 2025). Importantly, no studies to date have assessed adrenal responsiveness to standardized ACTH stimulation in this population, leaving key questions regarding physiological stress pathways during gender‐related minority stress.

The present study addresses this gap by evaluating basal adrenal hormones and ACTH‐stimulated cortisol and DHEAS responses in TGD adolescents referred for clinical evaluation, compared with age‐matched controls. By integrating developmental psychobiology with minority stress and embodiment frameworks, this exploratory work aims to clarify how psychosocial stressors may influence adrenal function during adolescence and to identify potential biomarkers relevant to health disparities among gender‐diverse youth. This design offers a complementary perspective to field‐based studies of diurnal cortisol by probing adrenal responsiveness under standardized endocrine challenge conditions, rather than relying solely on naturally occurring variation in daily stressors.

## 2 Methods

### 2.1 Study Design and Participants

This retrospective, case–control, single‐center study was conducted at the Institute for Maternal and Child Health—IRCCS “Burlo Garofolo” (Trieste, Italy), a tertiary university pediatric hospital. The study period spanned from January 2019 to October 2024.

#### 2.1.1 TGD Group (Cases)

We included all consecutive adolescents self‐identifying as TGD who underwent a standard dose synacthen test (SDST) as part of their evaluation at the Pediatric Gender Diversity Clinic (APEVAGE) (Roia et al. 2025), a multidisciplinary gender clinic where the SDST is routinely performed to assess general adrenal function (Wall et al. 2021). No participant had received prior endocrine therapy (e.g., pubertal blockers, gender‐affirming hormones, or menstrual suppression). Gender identity was assessed in routine clinical practice through a structured clinical interview using a two‐step method (current gender identity and sex assigned at birth), consistent with international recommendations (Bauer et al. 2017; Puckett et al. 2020). Based on clinical records, adolescents were identified as trans boys, trans girls, or nonbinary. Distress related to gender identity was not systematically assessed using standardized instruments and therefore could not be included as a variable in the present analysis.

#### 2.1.2 Control Group

Controls were adolescents referred for suspected nonclassical congenital adrenal hyperplasia (CAH), later excluded based on a 60‐min 17‐hydroxyprogesterone (17‐OHP) < 10 ng/mL (Speiser et al. 2018). The clinical indication was premature pubarche (Kurtoğlu and Hatipoğlu 2017). Controls were not evaluated for gender identity because this is not part of the routine assessment for premature pubarche; they were presumed cisgender based on the absence of indicators to the contrary in clinical records; this proxy classification limits direct inferences about minority stress in the control group. None reported psychological distress in their medical files. Unlike TGD youth, SDST was performed in controls exclusively for diagnostic exclusion of nonclassical CAH.

Demographic and anthropometric data were extracted from medical records: age, sex assigned at birth, height, target height, and BMI standard deviation scores (SDS) according to Italian growth charts (Cacciari et al. 2006).

### 2.2 SDST

The SDST evaluates adrenal cortical responsiveness to exogenous ACTH, providing an index of adrenal reserve under standardized pharmacological stimulation. All tests were performed at 8:00 a.m. by venipuncture. An intravenous bolus of 250 µg tetracosactide acetate (Synacthen, Alfasigma, Italy) was administered following clinical guidelines (Penco et al. 2021). Blood samples were collected at baseline (0 min) and 60 min poststimulation. The 30‐min sample was omitted due to limited additional diagnostic value in nonclassical CAH (Butt et al. 2020). Analytes measured included basal cortisol, DHEAS, 17‐OHP, and ACTH, as well as 60‐min cortisol and 17‐OHP.

### 2.3 Hormonal Assays

Cortisol levels were measured at 8:00 a.m. using the Access Cortisol kit on the Beckman Coulter DxI‐800 Analyzer. The assay demonstrated an analytical sensitivity of 11 nmol/L. The total imprecision (coefficient of variation [CV]) was below 12% for cortisol concentrations of approximately 138 nmol/L and below 10% for higher concentrations. DHEAS levels were measured using the Access DHEA‐S kit on the Beckman Coulter DxI‐800 Analyzer. The assay demonstrated an analytical sensitivity of < 2 µg/dL (< 53.22 nmol/L), with a total imprecision (CV) below 10% for concentrations ≥ 20 µg/dL (≥ 532.2 nmol/L). DHEAS values were converted from µg/dL to nmol/L (1 µg/dL = 26.61 nmol/L). Adrenocorticotropic hormone (ACTH) levels were determined using the LIAISON ACTH kit on the LIAISON XL Analyzer (DiaSorin). The assay's analytical sensitivity was 1.6 pg/mL, with a total imprecision (CV) below 10%. 17‐OHP levels were measured using a solid‐phase enzyme‐linked immunosorbent assay (ELISA) kit (DRG International kit) on the DSX Instrument from Dynex Technologies. The ELISA method is based on competitive binding; precision was assessed using four patient samples covering the measuring range in a single run with 10 replicates, yielding a mean CV < 10%. As part of good laboratory practice, calibration curves and controls were included in each analytical session for cortisol, DHEAS, ACTH, and 17‐OHP.

### 2.4 Statistical Analysis

Statistical analyses were performed using Jamovi (version 2.3.28.0). Descriptive statistics (absolute and percentage frequencies, medians, and interquartile range [IQR]) were used to summarize the data. Categorical variables were compared using the chi‐square test, while continuous variables were analyzed with the Kruskal–Wallis test for nonparametric data. Spearman's rank correlation was used to assess relationships between continuous variables. Multivariate linear regression was employed to examine associations between the dependent variable and multiple predictors, adjusting for potential confounders. All tests were two‐tailed, with statistical significance set at *p* < 0.05.

Because psychological distress and minority stressors were not assessed using standardized tools, they could not be included as adjustment variables. To mitigate potential biases, including selection bias from the single‐center design, measurement variability, and unmeasured confounders (e.g., psychological stress, social support, mental health comorbidities), we employed standardized protocols, multivariate adjustments, and age‐matched controls to enhance study validity.

The sample size was determined based on the retrospective data available at our center during the study period. A sensitivity power analysis was conducted to evaluate the minimum detectable effect sizes given the available sample (TGD *n* = 48; age‐matched controls *n* = 96). With *α* = 0.05 (two‐tailed), the study had 80% power to detect between‐group differences of Cohen's *d* ≈ 0.51. For correlation analyses within controls (*n* = 298), the detectable effect size was |*r*| ≈ 0.16. In multiple regression models including the TGD indicator and covariates, the detectable incremental effect was *f* ^2^ ≈ 0.018 (partial *R* ^2^ ≈ 1.8%). Using median and IQR values, approximate effect sizes for the main outcomes were computed: serum cortisol (*d* = 0.58), DHEAS‐to‐cortisol ratio (*d* = −0.51), and Δcortisol (*d* = −0.77). These values exceed the minimum detectable thresholds, supporting that the study was sufficiently powered for the primary comparisons.

### 2.5 Ethics

The study complied with STROBE guidelines for observational research (von Elm et al. 2014). Ethical Committee approval was not required per General Authorization to Process Personal Data for Scientific Research Purposes (Authorization no. 9/2014), which states that retrospective archive studies using anonymized ID codes that prevent direct identification do not require ethics approval (The Italian Data Protection Authority 2014). Parental informed consent was obtained during the initial visit, authorizing the secondary use of clinical data for research, epidemiological studies, pathology analysis, and training to enhance knowledge, care, and prevention. All data were collected in an anonymized database.

## 3 Results

### 3.1 Sample Characteristics

During the study period, data were available from 346 individuals, including 48 TGD adolescents and 298 controls. Complete datasets were obtained for most variables, with minor missing data for ACTH (*n* = 13) and target height (*n* = 52).

Compared with controls, TGD adolescents were significantly older (median 16.1 vs. 9.8 years; *p* < 0.001), more frequently assigned male at birth (35% vs. 19%; *p* = 0.009), and had lower height SDS (0.26 vs. 0.71; *p* = 0.035). No group differences were observed for BMI SDS or distance from target height. Group characteristics are reported in Table 1.

**TABLE 1:** Sample characteristics of TGD adolescents and controls.

	TGD individuals (*n *= 48)	Controls (*n *= 298)	*p*
Males (assigned sex at birth), *n* (%)	17 (35%)	56 (19%)	**0.009**
Age, years	16.1 (15.1; 17.0)	9.8 (8.0; 15.1)	**< 0.001**
Height, SDS	0.26 (−0.45; 1.13)	0.71 (−0.10; 1.54)	**0.035**
Target height, SDS	0.32 (−0.18; 0.77)	0.24 (−0.40; 1.00)	0.872
Height—target height, SDS	0.27 (−0.74; 0.58)	0.35 (−043; 1.05)	0.211
BMI, SDS	0.27 (−0.48; 1.10)	0.51 (−0.21; 1.27)	0.186
DHEAS, nmol/L	231 (146; 293)	142 (74; 222)	**< 0.001**
Cortisol, nmol/L	293 (233; 416)	214 (158; 309)	**< 0.001**
DHEAS‐to‐cortisol ratio	0.72 (0.46; 1.08)	0.59 (0.33; 1.12)	0.287
ACTH, pg/mL	21.4 (15.1; 32.7)	19.5 (15.0; 26.9)	0.261
17‐OH‐progesterone, ng/mL	1.6 (0.9; 2.4)	1.1 (0.7; 1.4)	**< 0.001**
Peak 17‐OH‐progesterone, ng/mL	3.6 (2.4; 4.7)	3.0 (2.4; 4.6)	0.519
Peak cortisol, nmol/L	674 (599; 748)	677 (608; 752)	0.766
Δcortisol, nmol/L	354 (265; 436)	446 (369; 532)	**< 0.001**

*Note:* Demographic and auxological variables in transgender and gender‐diverse (TGD) adolescents (*n* = 48) and controls (*n* = 298), with age‐matched subsample analysis (TGD *n* = 48; controls *n* = 96). Values are presented as median (interquartile range, IQR) or *n* (%). Variables include age (years), sex assigned at birth (male%), height SDS, distance from target height SDS, and BMI SDS. Significant *p* values are highlighted in bold.

### 3.2 Hormonal Differences

At baseline, both cortisol and 17‐OHP concentrations were significantly higher in TGD adolescents than in controls (293 vs. 214 nmol/L and 1.6 vs. 1.1 ng/mL, respectively; *p* < 0.001). Basal DHEAS levels were also elevated in TGD youth (231 vs. 142 nmol/L; *p* < 0.001), although the DHEAS‐to‐cortisol ratio did not differ significantly between groups.

Following stimulation, peak cortisol values were similar between groups, but the cortisol increment (Δcortisol) was significantly attenuated in TGD youth (354 vs. 446 nmol/L; *p* < 0.001), suggesting a blunted adrenal response. Peak 17‐OHP levels did not differ significantly.

**FIGURE 1:**
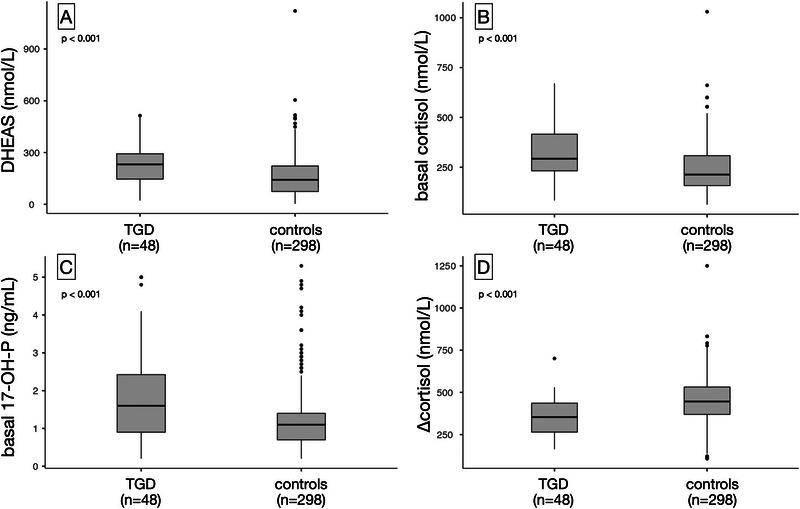
Baseline and stimulated hormone levels in TGD youth and controls. Group comparisons of (A) basal DHEAS (nmol/L), (B) basal cortisol (nmol/L), (C) basal 17‐hydroxyprogesterone (17‐OHP, ng/mL), and (D) cortisol increment following standard dose synacthen test (Δcortisol, nmol/L). TGD = transgender and gender‐diverse adolescents (*n* = 48); controls = adolescents evaluated for premature pubarche with CAH excluded (*n* = 298). Boxplots represent median and interquartile range. *p < 0.05; **p < 0.01; **p < 0.001.

Thus, TGD adolescents exhibited a profile characterized by higher basal cortisol but a smaller cortisol increment after stimulation, alongside an unchanged basal DHEAS‐to‐cortisol ratio in the full sample. Hormonal distributions are shown in Figure 1.

### 3.3 Correlation Analyses in Controls

Within the control group (*n* = 298), expected developmental patterns emerged: DHEAS and the DHEAS‐to‐cortisol ratio correlated strongly with age (*ρ* = 0.703 and *ρ* = 0.560; both *p* < 0.001) and positively with BMI SDS. Basal 17‐OHP was associated with age, sex assigned at birth, and height SDS, while Δcortisol correlated positively with BMI SDS. Basal cortisol showed no significant associations. Correlation coefficients are summarized in Table 2.

**TABLE 2:** Correlation analyses in controls (*n* = 298).

	DHEAS	Basal cortisol	DHEAS‐to cortisol ratio	Basal 17‐OH‐P	Δcortisol
Age	*ρ* = 0.703 ***p* < 0.001**	*ρ* = 0.110 *p* = 0.058	*ρ* = 0.560 ***p* < 0.001**	*ρ* = 0.497 ***p* < 0.001**	*ρ* = −0.058 *p* = 0.321
Male (assigned sex at birth)	*ρ* = −0.069 *p* = 0.234	*ρ* = −0.011 *p* = 0.853	*ρ* = −0.061 *p* = 0.292	*ρ* = −0.154 ***p* = 0.008**	*ρ* = −0.071 *p* = 0.212
Height SDS	*ρ* = −0.088 *p* = 0.129	*ρ* = −0.002 *p* = 0.068	*ρ* = −0.072 *p* = 0.212	*ρ* = −0.138 ***p* = 0.017**	*ρ* = −0.058 *p* = 0.321
BMI SDS	*ρ* = 0.198 ***p* < 0.001**	*ρ* = −0.090 *p* = 0.122	*ρ* = 0.204 ***p* < 0.001**	*ρ* = 0.014 *p* = 0.814	*ρ* = 0.237 ***p* < 0.001**

*Note:* Spearman's rank correlations (*ρ*) between adrenal hormones and demographic/auxological variables in the control group. Variables include age (years), sex assigned at birth, height SDS, and BMI SDS. Hormonal measures: basal DHEAS (nmol/L), DHEAS‐to‐cortisol ratio, basal 17‐hydroxyprogesterone (17‐OHP, ng/mL), Δcortisol (nmol/L), and basal cortisol (nmol/L). Significant *p* values are highlighted in bold.

### 3.4 Multivariate Analysis

**FIGURE 2:**
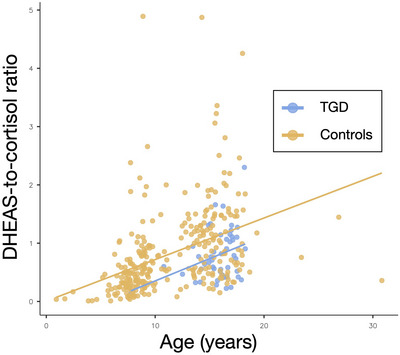
Multivariate regression results for TGD status. Standardized regression coefficients (*β*) and 95% confidence intervals for associations between TGD status and adrenal outcomes after adjustment for age, sex assigned at birth, height SDS, and BMI SDS (*n* = 346). Significant associations were observed for basal cortisol, Δcortisol, and DHEAS‐to‐cortisol ratio.

In regression models adjusting for age, sex assigned at birth, height SDS, and BMI SDS, TGD status remained independently associated with higher basal cortisol (*β* = 57.7, *p* = 0.006), lower Δcortisol (*β* = −58.6, *p* = 0.008), and a reduced DHEAS‐to‐cortisol ratio (*β* = −0.35, *p* = 0.001). Associations with DHEAS and basal 17‐OHP were not significant. The results are illustrated in Figure 2.

**FIGURE 3:**
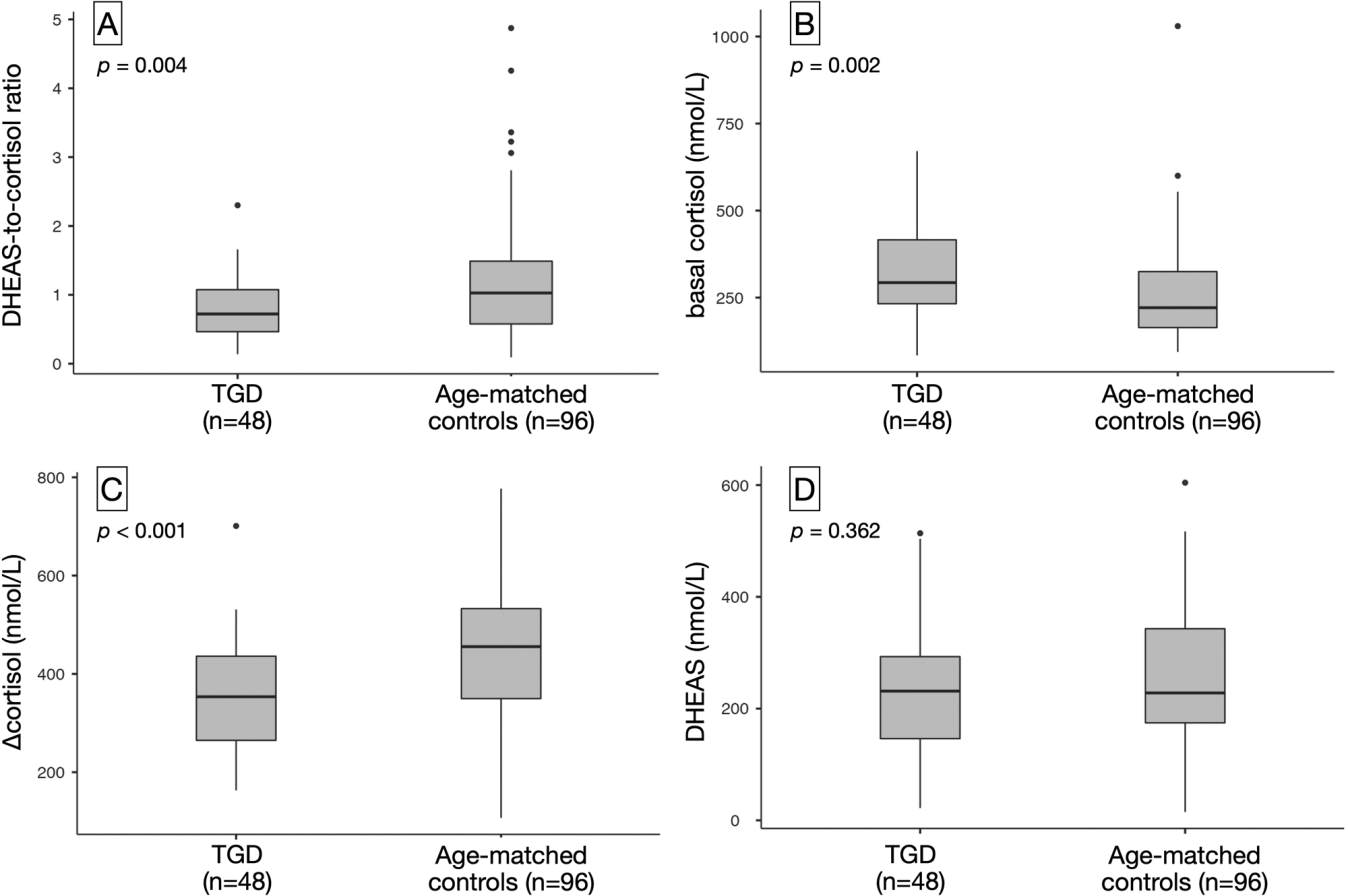
Age‐matched comparison of adrenal function between TGD youth and controls. Hormonal outcomes in TGD adolescents (*n* = 48) compared with 1:2 age‐matched controls (*n* = 96). (A) DHEAS‐to‐cortisol ratio, (B) basal cortisol (nmol/L), (C) Δcortisol (nmol/L), and (D) basal DHEAS (nmol/L). Matching minimized age‐related confounding. TGD youth showed higher basal cortisol and lower Δcortisol and DHEAS‐to‐cortisol ratios, consistent with altered adrenal responsiveness.

Because of significant age differences between groups, we performed a 1:2 age‐matched analysis (TGD:controls). The findings were consistent: compared with matched controls, TGD youth displayed a significantly lower DHEAS‐to‐cortisol ratio (0.72 vs. 1.03; *p* = 0.004), higher basal cortisol (293 vs. 221 nmol/L; *p* = 0.002), and reduced Δcortisol (+354 vs. +456 nmol/L; *p* < 0.001) (Table 3). Basal DHEAS did not differ between groups. These comparisons are shown in Figure 3.

**TABLE 3:** Characteristics of transgender and gender diverse (TGD) individuals compared to age‐matched control subjects (1:2 ratio).

	TGD individuals (*n* = 48)	Controls (*n* = 96)	*p*
Males (assigned sex at birth), *n* (%)	17 (35%)	5 (5%)	**< 0.001**
Age, years	16.1 (15.1; 17.0)	15.9 (15.1; 16.8)	0.632
Height, SDS	0.26 (−0.45; 1.13)	0.09 (−0.46; 1.07)	0.854
Target height, SDS	0.32 (−0.18; 0.77)	0.24 (−0.29; 0.75)	0.823
Height—target height, SDS	0.27 (−0.74; 0.58)	−0.17 (−0.66; 0.38)	0.273
BMI, SDS	0.27 (−0.48; 1.10)	0.63 (−0.40; 1.61)	0.158
DHEAS, nmol/L	231 (146; 293)	228 (175; 343)	0.362
Cortisol, nmol/L	293 (233; 416)	221 (164; 325)	**0.002**
DHEAS‐to‐cortisol ratio	0.72 (0.46; 1.08)	1.03 (0.58; 1.49)	**0.004**
ACTH, pg/mL	21.4 (15.1; 32.7)	20.2 (14.9; 27.1)	0.405
17‐OH‐progesterone, ng/mL	1.6 (0.9; 2.4)	1.3 (1.1; 1.9)	0.266
Peak 17‐OH‐progesterone, ng/mL	3.6 (2.4; 4.7)	3.4 (2.6; 5.0)	0.961
Peak cortisol, nmol/L	674 (599; 748)	683 (613; 775)	0.417
Δcortisol, nmol/L	354 (265; 436)	456 (350; 533)	**< 0.001**

*Note:* Data are presented as median (interquartile range) for continuous variables or as numbers (percentage) for categorical variables. Significant *p* values are highlighted in bold.

## 4 Discussion

### 4.1 Main Findings

This study examined adrenal stress‐related biochemical markers in TGD adolescents, providing new insights into HPA axis functioning during adolescence. Compared with age‐matched controls, TGD youth displayed elevated basal cortisol, a reduced DHEAS‐to‐cortisol ratio, and a blunted cortisol response to standardized ACTH stimulation (SDST). Together, these findings suggest alterations in HPA axis dynamics consistent with chronic stress exposure and with patterns of HPA axis dysregulation described in minority stress and allostatic load frameworks (Meyer 2003; McEwen 1998; Juster et al. 2010; Krieger 2010; DuBois et al. 2017; DuBois and Juster 2022; DuBois et al. 2024).

Elevated basal cortisol levels in the TGD group reflect sustained tonic activation of the HPA axis, potentially driven by persistent psychosocial stressors. Minority stress theory, which posits that marginalized populations experience chronic stress due to stigma, discrimination, and social rejection, provides a framework for understanding these findings (Meyer 2003; Hendricks and Testa 2012; Puckett et al. 2014). Consistent with previous research on transgender adults and sexual and gender minority samples (DuBois et al. 2017; DuBois and Juster 2022; Zoccola et al. 2017; DuBois et al. 2024; Figueroa et al. 2021) and other populations facing psychosocial adversity (Walsh et al. 2013; Rizk et al. 2018; Lewitzka et al. 2017), elevated cortisol may represent a biological embedding of social stress (Krieger 2010; McEwen 1998). However, in the present study, TGD status serves only as an indirect proxy for exposure to minority stressors, which were not directly measured; and therefore, causal inferences regarding specific stress experiences cannot be drawn. Prolonged HPA axis activation contributes to allostatic load (McEwen 1998), with downstream effects on metabolic, cognitive, and emotional systems (Chrousos 2009; Rosmond 2005).

Interestingly, despite similar peak cortisol levels between groups, TGD adolescents showed a significantly blunted increase (Δcortisol) in response to SDST. This attenuation is a hallmark of chronic stress and reflects diminished adrenal reactivity to novel challenges (Lovallo et al. 2000; Bunea et al. 2017). While such downregulation may protect against excessive glucocorticoid exposure, it potentially compromises stress responsiveness and increases vulnerability to psychopathology (O'Connor et al. 2017; et al. 2019). This pattern—elevated basal cortisol coupled with reduced dynamic reactivity—is consistent with HPA axis dysregulation observed in chronic stress populations (Lovallo et al. 2000; Bunea et al. 2017; O'Connor et al. 2017; Padden Padden et al. 2019; Schär et al. 2022) and in samples exposed to structural or identity‐related stigma (Juster et al. 2010; DuBois et al. 2017; DuBois and Juster 2022).

DHEAS levels, by contrast, did not differ significantly between the TGD and control groups, indicating that the lower DHEAS‐to‐cortisol ratio in TGD adolescents is primarily driven by elevated cortisol. The ratio serves as a sensitive index of HPA axis balance, with DHEAS exerting neuroprotective, anti‐glucocorticoid, and mood‐stabilizing effects (Kroboth et al. 1999; Tsigos and Chrousos 2002). Reduced ratios have been linked to cognitive deficits and affective disturbances in clinical populations (Andrisani et al. 2017). Furthermore, studies of anorexia nervosa patients report persistent hypothalamic dysfunction associated with low DHEAS‐to‐cortisol ratios (Andrisani et al. 2017). In TGD adolescents, Schagen et al. (2018) reported normal DHEAS levels with increases after GnRHa treatment, although no cortisol data were available. Our findings add to this literature by demonstrating that before medical intervention, TGD adolescents may show an altered DHEAS‐to‐cortisol balance, potentially reflecting neuroendocrine adaptation to minority stress and highlighting the value of considering both glucocorticoid and adrenal androgen pathways in studies of gender‐related stress (Kroboth et al. 1999; Gallagher and Young 2002; Buford and Willoughby 2008; Andrisani et al. 2017; Schagen et al. 2018; DuBois et al. 2024).

### 4.2 Developmental and Clinical Implications

Adolescence represents a critical period for neuroendocrine plasticity, where dysregulation of the HPA axis can exert lasting effects on emotional regulation, cognitive development, and metabolic health. The observed changes in TGD youth may constitute a biological signature of minority stress, reflecting the embodiment of social adversity (Krieger 2010; Brooks 1981; Meyer 2003; Hendricks and Testa 2012). Given the lack of standardized measures of distress or minority stressors, this interpretation should be viewed as hypothesis‐generating rather than definitive. Given that participants had not begun gender‐affirming medical treatments, the stress‐related biochemical profile likely reflects psychosocial, rather than medical, influences.

Clinically, these findings highlight the importance of early supportive interventions that reduce minority stress—such as family support, inclusive policies, and access to gender‐affirming care—as potential methods to mitigate HPA axis dysregulation (de Vries et al. 2014). Moreover, biomarkers such as Δcortisol and the DHEAS‐to‐cortisol ratio hold promise as objective indicators for monitoring the physiological impact and efficacy of psychosocial and medical interventions (Juster et al. 2010; DuBois and Juster 2022), although they should be interpreted as group‐level markers of risk rather than individual diagnostic tools. These questions are particularly salient in the current sociopolitical climate, in which TGD youth are frequently the focus of public debate and policy restrictions, with potential implications for both stress exposure and access to protective resources (White Hughto et al. 2015).

### 4.3 Strengths and Limitations

This study is among the first to use standardized endocrine challenge testing to assess adrenal function in TGD adolescents, enhancing confidence in the findings. The use of an adequately powered, age‐matched control group and rigorous biochemical protocols further strengthens the evidence.

Limitations include the retrospective, single‐center design, which limits generalizability. Controls, drawn from adolescents evaluated for endocrinological concerns unrelated to stress, may not perfectly represent a healthy population. Although controls were not referred for stress‐related concerns, they had endocrine indications (premature pubarche) that may differ from community samples (Kurtoğlu and Hatipoğlu 2017), potentially limiting generalizability. Although analyses controlled for age, BMI, and sex assigned at birth, unmeasured confounders such as social support, psychological stress, and mental health comorbidities were not evaluated, and no standardized measures of minority stress or gender‐related distress were available (Hendricks and Testa 2012; Puckett et al. 2014; DuBois et al. 2017). Ethical constraints precluded the use of truly healthy controls for ACTH stimulation.

In addition, the relatively small sample size restricted subgroup analyses and limited assessment of sex differences in HPA axis function, which have been described in broader pediatric populations (Hollanders et al. 2017). Future studies with larger samples will be required to examine potential interactions between sex assigned at birth, pubertal stage, and TGD status in relation to HPA axis functioning (Shirtcliff et al. 2009; Powers and Casey 2015; Biro et al. 2014). Importantly, no simultaneous neuropsychological assessments were conducted for this study, limiting the correlation between physiological findings and clinical symptoms.

The biomarkers assessed, although informative, do not capture the full complexity of stress responses in real‐world settings. The SDST probes adrenal responsiveness to pharmacological ACTH stimulation rather than to experiential or psychosocial stressors, and therefore provides complementary but partial information about stress biology (Speiser et al. 2018; McEwen 1998). Finally, the cross‐sectional nature of the study prevents causal inferences regarding stress exposure and neuroendocrine adaptations (Juster et al. 2010).

### 4.4 Future Directions

Future research should prioritize longitudinal, multimodal studies integrating psychosocial assessments, neuroendocrine biomarkers, and clinical outcomes to elucidate causal pathways and mechanisms underlying stress biology in TGD youth (Juster et al. 2010; DuBois and Juster 2022; DuBois et al. 2024). Such studies should incorporate standardized measures of minority stress, gender‐related distress, and protective factors such as social support and gender euphoria (Hendricks and Testa 2012; Puckett et al. 2014; de Vries et al. 2014; White Hughto et al. 2015).

Investigations into sex‐specific HPA axis responses are warranted, as puberty and gonadal hormones likely modulate stress physiology. The development and validation of sensitive biomarkers, such as Δcortisol and DHEAS‐to‐cortisol ratios, may support personalized monitoring of therapeutic interventions.

Together, these efforts will enhance the understanding of minority stress embodiment, inform tailored care strategies, and ultimately improve health outcomes for TGD adolescents.

## 5 Conclusions

This study provides preliminary evidence of altered HPA axis functioning in TGD adolescents, characterized by elevated basal cortisol, reduced DHEAS‐to‐cortisol ratios, and blunted cortisol responses to pharmacological stimulation. These findings are consistent with the biological embedding of minority stress during adolescence, a sensitive developmental window for stress regulation, although TGD status in this study serves only as an indirect proxy for exposure to minority stressors. While exploratory, our results highlight the need for longitudinal studies that integrate psychosocial, neuroendocrine, and developmental outcomes to clarify the pathways linking minority stress and protective factors to health disparities. Such research may inform interventions aimed at fostering supportive environments and reducing physiological stress burden in gender‐diverse youth. Identifying objective biomarkers of minority stress may ultimately guide interventions aimed at improving both mental and physical health outcomes in gender‐diverse adolescents.

## Author Contributions

**Simone Coslovich**: writing – review and editing, writing – original draft, visualization, project administration, methodology, investigation, data curation, conceptualization. **Stefania Tonetto**: writing – review and editing, writing – original draft, visualization, project administration, methodology, investigation, data curation, conceptualization. **Giulia Bragato**: writing – review and editing, writing – original draft, data curation, conceptualization. **Gianluca Tamaro**: writing – review and editing, writing – original draft, supervision. **Alice Fachin**: writing – review and editing, writing – original draft, supervision. **Paolo Dalena**: writing – review and editing, formal analysis. **Lorenzo Zandonà**: writing – review and editing, formal analysis. **Antonella Fabretto**: writing – review and editing, formal analysis. **Egidio Barbi**: writing – review and editing, supervision, methodology. **Gianluca Tornese**: writing – review and editing, supervision, methodology, investigation, funding acquisition, conceptualization.

## Funding

This work was supported by the Italian Ministry of Health, Italy, through the contribution given to the Institute for Maternal and Child Health IRCCS Burlo Garofolo, Trieste, Italy (RC 6/25).

## Conflicts of Interest

The authors declare no conflicts of interest.
